# Valores de Referência Ecocardiográficos: Por que o Brasil Precisa Falar com a Própria Voz

**DOI:** 10.36660/abc.20260155

**Published:** 2026-04-23

**Authors:** Imara Correia de Queiroz Barbosa, Israel Nilton de Almeida Feitosa

**Affiliations:** 1 Universidade Federal de Campina Grande Campina Grande PB Brasil Universidade Federal de Campina Grande (UAMED), Campina Grande, PB – Brasil; 2 Ebserh (Empresa Brasileira de Serviços Hospitalares) Campina Grande PB Brasil Ebserh (Empresa Brasileira de Serviços Hospitalares), Campina Grande, PB – Brasil

**Keywords:** Ecocardiografia, Valores de Referência, Strain Longitudinal Global

As medidas ecocardiográficas são pilares do diagnóstico, da estratificação de risco e da tomada de decisão terapêutica em diversas condições cardiovasculares. Apesar disso, a prática clínica brasileira ainda depende, em grande parte, de intervalos de referência derivados de populações europeias e norte-americanas. Em um país de dimensões continentais, marcado por ampla heterogeneidade étnica, antropométrica e socioeconômica, essa extrapolação levanta questionamentos legítimos sobre precisão diagnóstica e equidade na avaliação cardiovascular.

Nesta edição dos Arquivos Brasileiros de Cardiologia, Rodrigues et al.^1^ apresentam um estudo oportuno e metodologicamente consistente, estabelecendo valores de referência contemporâneos para parâmetros ecocardiográficos em adultos brasileiros saudáveis. A inclusão de 496 voluntários provenientes de 20 laboratórios distribuídos pelas cinco regiões do país confere ao trabalho uma representatividade inédita no cenário nacional e enfrenta uma lacuna histórica da cardiologia brasileira.^1^

Entre os principais méritos do estudo destaca-se a padronização rigorosa da aquisição das imagens e a análise centralizada, independente de fabricante, conduzida por investigadores experientes. Os resultados confirmam diferenças consistentes relacionadas ao sexo: homens apresentaram maiores dimensões, volumes e massa do ventrículo esquerdo, mesmo após indexação pela superfície corporal, enquanto mulheres demonstraram fração de ejeção discretamente mais elevada e valores significativamente superiores de deformação miocárdica, incluindo strain longitudinal global dos ventrículos esquerdo e direito. Esses achados reforçam evidências de que mulheres apresentam mecânica sistólica mais eficiente e sublinham a necessidade de intervalos de referência específicos por sexo, particularmente para parâmetros baseados em strain, cada vez mais incorporados à prática clínica.^1^

**Figura 1:**
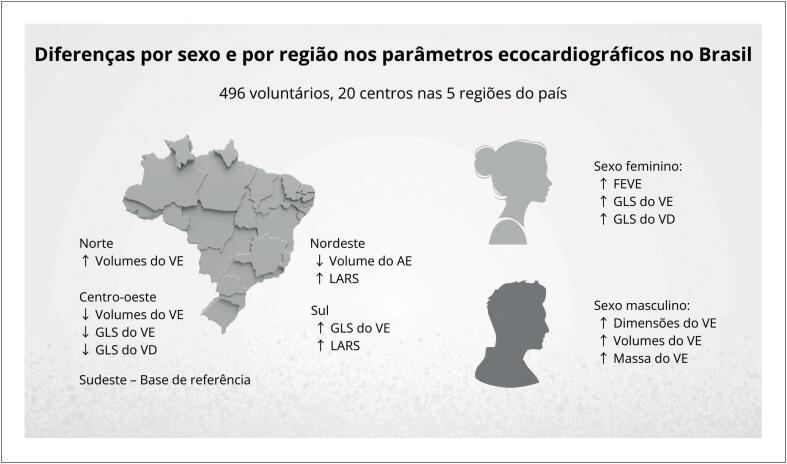
Variações relacionadas ao sexo e à região nas medidas ecocardiográficas observadas em uma coorte multicêntrica brasileira. AE: átrio esquerdo; LARS: strain de reservatório do átrio esquerdo; VE: ventrículo esquerdo; FEVE: fração de ejeção do ventrículo esquerdo; GLS do VE: strain longitudinal global do ventrículo esquerdo; GLS do VD: strain longitudinal global do ventrículo direito.

O estudo avança ainda ao demonstrar variabilidade regional nas medidas ecocardiográficas dentro do próprio Brasil (Figura 1). Diferenças nas dimensões das câmaras cardíacas e nos parâmetros de deformação miocárdica entre regiões, como menores valores de strain ventricular no Centro-Oeste e menores volumes do átrio esquerdo no Nordeste, sugerem que fatores demográficos, antropométricos e possivelmente ambientais influenciam o fenótipo cardíaco. Em uma população altamente miscigenada, tais achados reforçam a complexidade de se estabelecer limites universais de normalidade.^1^

Algumas limitações merecem menção. A predominância de participantes da região Sudeste, embora compatível com a distribuição populacional nacional, pode restringir comparações regionais mais equilibradas. A exclusão de indivíduos com obesidade, adequada para a definição de normalidade, limita a aplicabilidade direta a cenários clínicos contemporâneos. Além disso, a representação modesta de determinados grupos étnicos evidencia os desafios persistentes na construção de bases normativas plenamente inclusivas.

Embora um estudo brasileiro anterior tenha estabelecido valores de normalidade ecocardiográficos em uma população adulta assintomática, seu caráter unicêntrico limitou a abrangência nacional desses achados.^2^

Comparado às principais referências internacionais, a diretriz conjunta da American Society of Echocardiography e da European Association of Cardiovascular Imaging (ASE/EACVI), e o documento da British Society of Echocardiography (BSE), o estudo brasileiro adota uma abordagem conceitualmente distinta. As diretrizes ASE/EACVI baseiam-se na agregação de grandes coortes internacionais previamente publicadas, como Asklepios,^3^ Flemengho,^4^ CARDIA^5^ e NORRE,^6^ reunindo populações predominantemente europeias e norte-americanas. A definição de normalidade nesses documentos apoia-se em critérios clínicos e laboratoriais objetivos — incluindo pressão arterial, glicemia, perfil lipídico, creatinina e índice de massa corporal — com o objetivo de caracterizar uma população metabolicamente saudável.^6-8^

Entretanto, por se tratar da consolidação de bancos de dados independentes, essas diretrizes incorporam estudos com protocolos ecocardiográficos não inteiramente uniformes e sem garantia de aquisição padronizada ou análise centralizada em laboratório dedicado e independente, o que pode introduzir heterogeneidade metodológica inerente. De forma semelhante, o documento da BSE declara que seus intervalos de referência derivam majoritariamente do estudo NORRE, mantendo a lógica de padronização técnica e operacional a partir de populações europeias, com critérios de seleção também ancorados em variáveis clínicas e laboratoriais. Em contraste, o estudo brasileiro adota uma coorte nacional prospectiva, com aquisição padronizada e análise centralizada dentro de um único desenho metodológico, privilegiando a consistência interna e a representatividade clínica da população avaliada.^6-8^

Do ponto de vista prático, os limites superiores para dimensões e massa ventricular esquerda mostraram-se menores no Brasil, especialmente entre homens, o que pode aumentar a sensibilidade para a identificação precoce de dilatação e hipertrofia. Os volumes ventriculares indexados também tendem a ser ligeiramente inferiores aos descritos em diretrizes internacionais, enquanto há concordância quanto ao volume atrial esquerdo indexado e à fração de ejeção. Para o strain longitudinal global, os valores médios são semelhantes, mas o diferencial reside na robustez metodológica e na documentação da variabilidade regional.^1^

O estudo de Rodrigues et al.^1^ representa um avanço importante para que a ecocardiografia brasileira passe a se apoiar em referências construídas em seu próprio contexto populacional. Mais do que oferecer novos números, o trabalho convida à reflexão sobre como definimos normalidade e para quem esses limites realmente servem. Em um país marcado pela diversidade, a construção de referências próprias é não apenas desejável, mas necessária.
