# Supplementary material for: Valores de Referência Ecocardiográficos: Por que o Brasil Precisa Falar com a Própria Voz
Source: Arq Bras Cardiol. 2026 Apr 23;123(2):e20260155. [Article in Portuguese] doi: 10.36660/abc.20260155 (PMC13128172; doi:10.36660/abc.20260155)
Supplement: Translation

# Translation: Echocardiographic Reference Values: Why Brazil Needs to Speak With its Own Voice

**DOI:** 10.36660/abc.20260155i

**Authors:** Imara Correia de Queiroz Barbosa, Israel Nilton de Almeida Feitosa

**Affiliations:** 1 Campina Grande PB Brazil Universidade Federal de Campina Grande (UAMED), Campina Grande, PB – Brazil; 2 Campina Grande PB Brazil Ebserh (Empresa Brasileira de Serviços Hospitalares), Campina Grande, PB – Brazil

**Keywords:** Echocardiography, Reference Values, Global Longitudinal Strain

Echocardiographic measurements are cornerstones of diagnosis, risk stratification, and therapeutic decision-making across a wide range of cardiovascular conditions. Despite this, clinical practice in Brazil still relies largely on reference ranges derived from European and North American populations. In a country of continental dimensions, marked by substantial ethnic, anthropometric, and socioeconomic heterogeneity, such extrapolation raises legitimate concerns regarding diagnostic accuracy and equity in cardiovascular assessment.

In this issue of the *Arquivos Brasileiros de Cardiologia*, Rodrigues et al.^1^ present a timely and methodologically sound study establishing contemporary reference values for echocardiographic parameters in healthy Brazilian adults. The inclusion of 496 volunteers recruited from 20 echocardiography laboratories across the five major geographic regions of the country provides unprecedented national representativeness and addresses a longstanding gap in Brazilian cardiology.^1^

Among the main strengths of the study is the rigorous standardization of image acquisition and the centralized, vendor-independent analysis performed by experienced investigators. The results confirm consistent sex-related differences: men exhibited larger left ventricular dimensions, volumes, and mass, even after indexing to body surface area, whereas women demonstrated slightly higher left ventricular ejection fraction and significantly greater myocardial deformation values, including left and right ventricular global longitudinal strain. These findings reinforce accumulating evidence that women exhibit more efficient systolic mechanics and underscore the importance of sex-specific reference ranges, particularly for strain-based parameters that are increasingly incorporated into routine clinical practice.^1^

The study further advances the field by demonstrating regional variability in echocardiographic measurements within Brazil itself (Figure 1). Differences in chamber dimensions and myocardial deformation parameters across geographic regions—such as lower ventricular strain values in the Central-West and smaller left atrial volumes in the Northeast—suggest that demographic, anthropometric, and possibly environmental factors influence cardiac phenotypes. In a highly admixed population, these observations further highlight the complexity of defining universal limits of normality.^1^

**Figure 1:** Sex-related and regional variations in echocardiographic measurements observed in a multicenter Brazilian cohort. LA: left atrium; LARS: left atrial reservoir strain; LV: left ventricle; LVEF: left ventricular ejection fraction; LV GLS: left ventricular global longitudinal strain; RV GLS: right ventricular global longitudinal strain.

Some limitations deserve consideration. The predominance of participants from the Southeast region, although consistent with national population distribution, may have limited broader regional comparisons. The exclusion of individuals with obesity, while appropriate for defining normality, restricts direct applicability to contemporary clinical settings. In addition, the modest representation of certain ethnic groups underscores the persistent challenges in constructing fully inclusive normative databases.

Although a previous Brazilian study established echocardiographic normal reference values in an asymptomatic adult population, its single-center design limited the national applicability of these findings.^2^

When compared with major international references, the joint guidelines of the American Society of Echocardiography and the European Association of Cardiovascular Imaging (ASE/EACVI), and the document of the British Society of Echocardiography (BSE), the Brazilian study adopts a conceptually distinct approach. The ASE/EACVI guidelines are based on the aggregation of large, previously published international cohorts, such as Asklepios,^3^ Flemengho,^4^ CARDIA,^5^ and NORRE,^6^ comprising predominantly European and North American populations. In these documents, normality is defined using objective clinical and laboratory criteria, including blood pressure, glycemia, lipid profile, creatinine, and body mass index, aiming to characterize a metabolically healthy population.^6-8^

However, because these guidelines consolidate independent datasets, they incorporate studies with non-uniform echocardiographic protocols and without guaranteed standardized acquisition or centralized analysis in a dedicated, independent core laboratory, potentially introducing inherent methodological heterogeneity. Similarly, the BSE document states that its reference ranges are largely derived from the NORRE study, maintaining a framework centered on European populations and selection criteria anchored in clinical and laboratory variables. In contrast, the Brazilian study is based on a prospective national cohort, with standardized acquisition and centralized analysis within a single methodological design, prioritizing internal consistency and clinical representativeness of the evaluated population.^6-8^

From a practical standpoint, the upper limits for left ventricular dimensions and mass were lower in Brazil, particularly among men, potentially increasing sensitivity for the early detection of dilation and hypertrophy. Indexed ventricular volumes also tended to be slightly lower than those described in international guidelines, while concordance was observed for indexed left atrial volume and ejection fraction. For global longitudinal strain, mean values were comparable; however, the distinguishing features of the Brazilian study lie in its methodological robustness and in the documentation of regional variability.^1^

In summary, the study by Rodrigues et al.^1^ represents an important advance toward enabling Brazilian echocardiography to rely on reference values derived from its own population context. Beyond providing new numbers, the work invites reflection on how normality is defined and for whom these limits are intended. In a country characterized by diversity, the construction of population-specific reference values is not only desirable but necessary.
